# Irisin protects female mice with LPS-induced endometritis through the AMPK/NF-κB pathway

**DOI:** 10.22038/ijbms.2021.56781.12678

**Published:** 2021-09

**Authors:** Xi Jiang, Ying Hu, Yingjie Zhou, Jin Chen, Chonglu Sun, Ziwei Chen, Changfeng Jing, Lexing Xu, Fuhe Liu, Wenjuan Ni, Xuefeng Yu, Lei Chen

**Affiliations:** 1 Zhejiang University Mingzhou Hospital, Ningbo, 315000, China; 2 Department of Pharmacy, Zhejiang Pharmaceutical College, Ningbo, 315000, China

**Keywords:** AMP-activated protein – kinases, Endometritis, Inflammation, Irisin, Lipopolysaccharide, NF-κB

## Abstract

**Objective(s):**

This research was designed to determine the role of irisin in lipopolysaccharide (LPS)-induced endometritis in female mice.

**Materials and Methods:**

Animals were randomly assigned into sham, sham + irisin, LPS, LPS + irisin (0.1, 1, 10 μg/kg), and LPS + irisin + compound C groups. Histological features and expression of AMPK, NF-κB, inflammatory mediators, and oxidative stress markers were compared among different groups.

**Results:**

The results showed that LPS resulted in obvious uterus damage, meanwhile, the inflammatory mediators (COX-2, iNOS, IL-1β, IL-6, and TNF-α), as well as NF-κB in the uterine tissue, were significantly increased and the level of adenosine monophosphate-activated protein kinase (AMPK) was reduced. Nevertheless, pretreatment with irisin reversed the phenomena caused by LPS. Interestingly, compound C (AMPK inhibitor) abolished irisin’s effects on the uterus, which suggested that irisin’s beneficial function was achieved through regulating the AMPK-NF-κB pathway. Moreover, LPS-induced alterations of oxidative factors (MnSOD, GSH, and MDA) were reversed significantly by pretreatment with irisin. This data indicated irisin’s beneficial function was also related to antioxidation besides anti-inflammation.

**Conclusion:**

Our study implies that irisin is a potential therapeutic agent for endometritis.

## Introduction

Endometritis is a kind of primary infectious disease of the uterus leading to purulent inflammatory mucus secretion in the uterine of female mammals (1). The inflammation may induce disorder of the intrauterine environment and inability to maintain viable embryos, and eventually result in infertility (2). In animal husbandry, endometritis in cows leads to lower milk production, thereby causes huge economic losses to the farm (3). Antibiotics are the commonly used medicine in the treatment of endometritis; however, severe antibiotic resistance and drug residue restrict wide application (4). Therefore, more efforts should be made to seek a safe and effective strategy for endometritis treatment.

Irisin is a skeletal muscle-derived myokine secreted after physical exercise (5). Elevated irisin significantly increases energy expenditure through converting white adipose to brown fat (6). In the last decade, evidence proved irisin was also secreted into blood besides muscles, and the circulating irisin was demonstrated to exert beneficial effects in many diseases (7-9). The positive role of irisin is mainly associated with the adenosine monophosphate-activated protein kinase (AMPK) pathway (10). AMPK is an important factor regulating not only energy metabolism but also the development of inflammation, which was achieved by modulating the activity of nuclear factor-kappaB (NF-κB), a well-known inducer of inflammatory factors such as COX-2, iNOS, IL-1β, IL-6, and TNF-α (11). Previous data showed that irisin could suppress NF-κB p65 phosphorylation and decrease the levels of pro-inflammatory genes through the activation of AMPK in INS-1E cells in glucolipotoxic conditions (12). It was also documented that irisin plays an important role in neurodegenerative diseases by decreasing IL-1β, IL-6, and COX-2 in astrocytes (13). Similar to other inflammatory diseases, inflammatory factors including IL-6, IL-1β, and TNF-α were found to increase in the uterus in the endometritis condition (14). In addition, COX-2 and iNOS were also found to be increased in rats with endometritis (15). In view of the anti-inflammatory ability of irisin, we speculate that irisin may be beneficial to relieving endometritis. 

In this research, the lipopolysaccharide (LPS)-induced endometritis model was established in mice, and irisin’s function on endometritis was explored. The expression of AMPK, NF-κB, and inflammatory cytokines was measured to investigate the potential mechanism.

## Materials and Methods

***Animals***

A total of 70 female BALB/c mice weighing 30 g with an age of 8 weeks were acquired by the Experimental Animal Center of Wenzhou Medical University. Animals were housed in a cage under at room temperature and humidity of 50 ± 10%. All procedures in this study were approved (approval number: wydw2019-0138) by the Animal Care and Use Committee of Wenzhou Medical University. 

***Study design***

**Figure 1:**
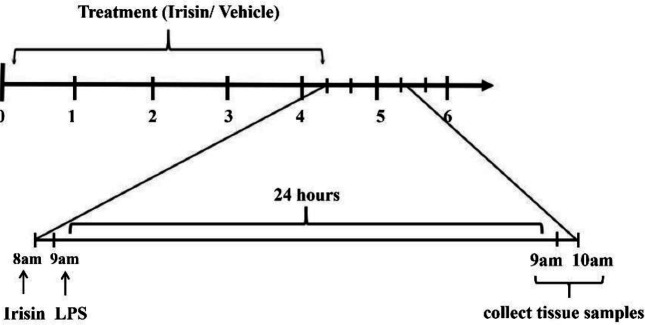
Treatment schedule. Mice received irisin treatment (0.1, 1, 10 μg/kg, IV) for 4 days. LPS was administrated on day 4 after irisin treatment. Mice were sacrificed 24 hr after LPS injection. Mice in the 10 μg/kg irisin-treated group simultaneously received 4 days of compound C (10 mg/kg, IP) treatment. LPS: lipopolysaccharide

According to Wang *et al*’s study, levels of inflammatory cytokines and oxidative stress markers were not significantly different among mice with different stages of the estrus cycle (16). Hence, the mice in this study were randomly assigned regardless of the estrus cycle. The mice were divided into 7 groups: sham, sham + irisin (10 μg/kg, IV), LPS, LPS + irisin (dose at 0.1, 1, 10 μg/kg, IV), and LPS + irisin (10 μg/kg, IV) + compound C (AMPK inhibitor) (10 mg/kg, IP) groups. Each group included 10 mice. The LPS-induced endometritis mouse model was established based on a previous study (17). Briefly, a mixed solution containing ketamine hydrochloride (50 mg/kg) and xylazine (5 mg/kg) was intraperitoneally injected into the mice. After making two lateral incisions 1 cm above the genitals, mice were injected with LPS (2.5 mg/ml) on each side of the uterus (20 μl/per side) except the sham group, the mice in which were injected with an equal volume of vehicle (normal saline) instead. Prior to LPS (Sigma-Aldrich) injection, mice in groups with irisin treatment were administrated irisin for 4 consecutive days, and mice in 10 μg/kg irisin-treated group also received 4 days of compound C treatment (Figure 1). All the mice were killed via **CO**_2_ inhalation 24 hr after LPS or vehicle injection. The uterine tissues of these mice were used for histological and biochemical analysis.

***Histological examination***

The uterine tissues were soaked in 4% paraformaldehyde for 48 hr and then embedded in paraffin. Afterward, the tissues were cut into slices with 5 µm each. The tissue sections were stained with hematoxylin and eosin dye solution. In the end, the stained tissues were observed via a microscope (Olympus, IX71, Tokyo, Japan).

***Biochemical analysis***

mRNA expressions of COX-2 and iNOS in the uterine tissue were determined by PCR. Protein levels of AMPK, COX-2, and iNOS in the uterine tissue were tested by western blot. The levels of NF-κB and inflammatory cytokines (IL-6, IL-1β, TNF-α) in the tissues, and irisin levels in the tissue and serum were detected by ELISA. The levels of oxidative stress markers (GSH, MDA, and SOD) in the tissues were detected by the biochemical method.

*Quantitative real-time PCR*

RNA kit (Bio-Rad. Labs) was used to prepare tissue samples. Total RNA was extracted by Trizol reagent (Trizol Invitrogen). The concentration of RNA was measured by a spectrophotometer (Bio-Rad. Labs). PCR reaction was achieved using an iCycler Real-Time PCR machine (Bio-Rad, Hercules CA, USA). Each sample was added with 50 nmol/l SYBR Green (iQ SYBR Green supermix reagent, Bio-Rad). Steps of real-time PCR: initial denaturation at 95 °C for 10 min, then 40 cycles at 95 °C for 10 sec, 58 °C for 30 sec. Finally, a melting curve was acquired by holding at 95 °C for 15 sec, cooling to 60 °C for 1 min, and then heating slowly at 0.5 °C / sec until 95 °C. The primer sequences of iNOS: forward (5′-CCTCCTCCACCCTACCAAGT-3′), reverse (5′-CACCCAAAGTGCTTCAGTCA-3′); COX-2: forward (5′-TGGGTGTGAAAGGAAATAAGGA-3′), reverse (5’-GAAGTGCTGG GCAAAGAATG-3’); β-actin: forward (5’-TGGAATCCTGTGGCATCCATGAAAC-3’), Reverse (5’-AAAACGCAGCTCAGTAACAGTCCG-3’).

*Western-blot analysis*

The protein concentration of each sample was tested by BCA kit (Thermo Scientific) and 40 μg protein was included in each band. Blots were blocked with milk for 2 hr after membrane transferring. Then, the blots were incubated with primary antibodies (anti-pAMPK, anti-AMPK, and anti-β-actin, purchased from Abcam; anti-COX-2 and anti-iNOS, obtained from Santa Cruz) and then secondary antibodies. Finally, images were taken by a fluorescence scanner, and the data were analyzed.

*ELISA*

The concentrations of NF-κBp65, IL-6, IL-1β, and TNF-α were evaluated by an ELISA kit (Thermo Scientific). The OD values of IL-6, IL-1β, and TNF-α were tested at 450 nm wavelength, while the OD value of NF-κBp65 was assessed at 405 nm wavelength. Irisin levels were measured with an ELISA kit purchased from Phoenix Pharmaceuticals, Burlingame, CA, USA. The minimum detectable irisin concentration was 1.29 ng/ml (18). Inter-assay (between days) and intra-assay (within days) values were 8–10% and 4–6%, respectively. The absorbance was read at 450 nm wavelength with an ELISA reader (ELX 800).

*Biochemical methods for detection of oxidative-stress markers*

Mn-superoxide dismutase (MnSOD). According to the cytochrome c reduction method reported by MacMillan-Crow and Thompson (18), The amount of enzyme which is essential to produce 50% inhibition was regarded as one unit and the results were expressed as U/mg protein.

Total glutathione (GSH) content. Mitochondrial GSH was measured by the method reported by Sadik *et al*. (18). In brief, an equal volume of 1% (w/v) sulfosalicylic acid was added to an aliquot of mitochondrial fraction, the mixture was centrifuged at 10,000g for 10 min. The supernatant was gathered and added with 0.4 M Tris buffer (pH 8.9) and 0.1 ml of 0.01 M 5,50-dithiobis-(2-nitrobenzoic acid) (DTNB). The absorbance was then detected at 412 nm wavelength. The data were expressed as μmol GSH/mg protein.

Lipid peroxidation (LPO). The LPO level was determined by detecting the pink chromophore generated by the reaction of thiobarbituric acid (TBARS) with malondialdehyde (MDA) at 535 nm wavelength (18). In brief, the tissue homogenate was incubated with 0.375 % thiobarbituric acid, 5 M HCl, and 15 % trichloroacetic acid at 95 °C for 15 min, and then cooled to room temperature and centrifuged for 10 min at 1,000g. The level of LPO was calculated as the concentration of malondialdehyde in nmol formed per milligram of protein.

***Data analysis***

Data analysis was performed by SPSS software (IBM, USA). One-way ANOVA was used to analyze the differences between groups. *Post-hoc* analysis was performed using the Duncan test. Data are expressed as mean ± SD. *P*<0.05 was regarded as statistical difference.

## Results

***Irisin reverses LPS-induced histopathological changes of the uterus***

**Figure 2:**
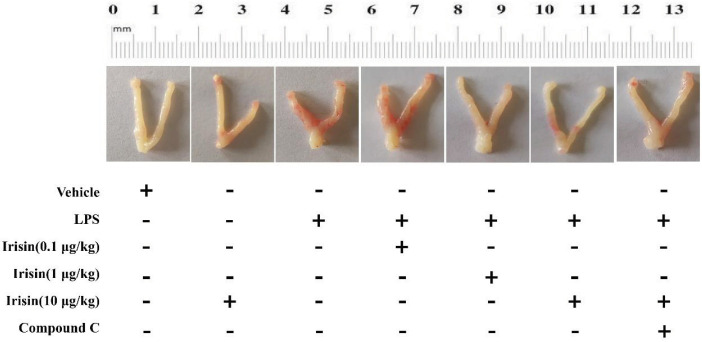
Effect of irisin on endometrial inflammation in LPS-challenged female mice. 10 μg/kg irisin treatment significantly attenuated LPS-induced redness and swelling of the uterus

As can be found in Figure 2, the uterus of the control group was smooth and elastic, while the uterus in the LPS group was markedly damaged with characteristics of congestion, edema, irregular shape, and poor toughness. Irisin in high dose (10 μg/kg) improved the damage of the uterus. Interestingly, the positive function of irisin on the uterus was partially reversed by compound C (Figure 2). 

**Figure 3:**
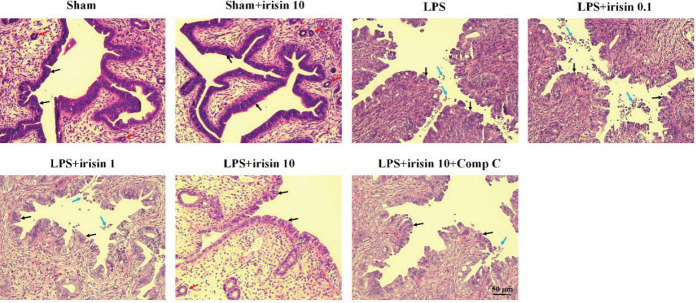
HE staining (200 ×) on the uterine tissue in sham, sham+irisin (10 μg/kg, IV), LPS, LPS+irisin (dose at 0.1, 1, 10 μg/kg, IV), LPS+irisin (10 μg/kg, IV) + compound C groups. The epidermal cells were normally arranged and clearly visible (black arrows), and endometrial glands (red arrows) could be observed in the sham group. LPS administration induced structural damage and epithelial shedding (blue arrows), and no endometrial glands could be clearly observed. Pretreatment with irisin (10 μg/kg) significantly attenuated LPS-induced histopathologic changes. The intact epithelium layer as indicated by black arrows and the epithelial cell exfoliation was indicated by blue arrows and the endometrial gland was indicated by red arrows

Results of the HE experiment were shown in Figure 3. The epidermal cells of the uterus were normally arranged and the cells in the tissue were clearly visible in the sham group. Besides, endometrial glands could be easily distinguished. However, after LPS administration, the uterine tissue was markedly damaged with features of hyperemia, hemorrhage, and shedding of epithelial cells. The outline of endometrial glands disappeared, and the alterations of endometrial epithelium height could be found. In addition, the uterine lumen was narrowed because of hyperemia and swelling (supplementary Figure 1). Pretreatment with irisin (10 μg/kg) significantly attenuated LPS-induced histopathologic changes. Whereas irisin’s improvement was partially abolished by AMPK inhibitor compound C. 

***Changes of irisin level in the uterus of LPS mice***

**Figure 4:**
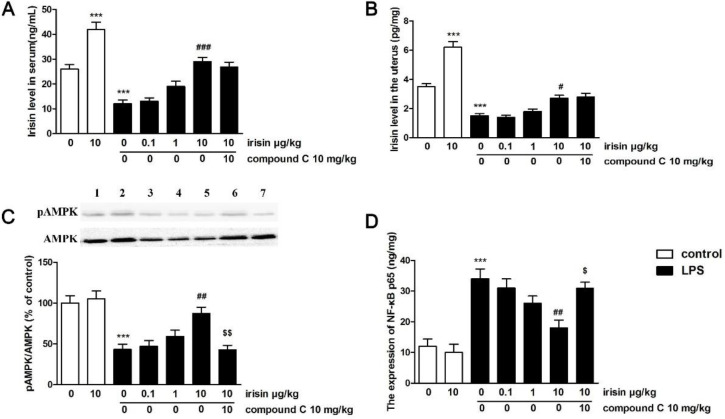
(A) Expression of irisin in the serum. (B) Expression of irisin in the uterine tissue. (C) Effect of irisin on pAMPK expression in the uterus. Lane 1: sham group; Lane 2: sham+irisin (10 μg/kg, IV) group; Lane 3: LPS group; Lane 4-6: LPS + irisin (0.1, 1, 10 μg/kg, IV)-treated groups; Lane 7: LPS + irisin (10 μg/kg, IV) + compound C group. (D) Effect of irisin on NF-κBp65 expression in the uterus. Values were the mean ± SEM (n=6 per group). *** *P*<0.001 vs the sham group; #*P*<0.05, ## *P*<0.01, and ### *P*<0.001 vs LPS group; $*P*<0.05 and $$P<0.01 vs LPS + irisin (10 μg/kg, IV) group

In LPS-treated mice, significant decreases of irisin expression were found in serum and uterine tissue (*P*<0.001 for serum and uterus, Figures 4A-B). Injection of exogenous irisin (10 μg/kg) markedly improved these situations (*P*<0.001 for serum, *P*<0.05 for uterus).

***Irisin increases AMPK and inhibits NF-κBp65 in the uterus of LPS-treated mice***

A significant decrease of pAMPK in the uterus (*P*<0.001, Figure 4C) was observed in LPS-treated mice. Irisin treatment (10 μg/kg) obviously reversed this reduction (*P*<0.01). Compound C suppressed the activation of AMPK triggered by irisin in the uterus (*P*<0.01).

Overexpression of NF-κBp65 stimulated by LPS was found in the uterus (*P*<0.001, Figure 4D). Irisin treatment at the concentration of 10 μg/kg greatly suppressed the overexpression of NF-κBp65 (*P*<0.01). Nevertheless, the restoration of abnormal NF-κBp65 expression induced by irisin in the uterus was abolished by compound C co-treatment (*P*<0.05).

***Irisin inhibits overexpression of inflammatory mediators in the uterus of LPS-treated mice***

**Figure 5:**
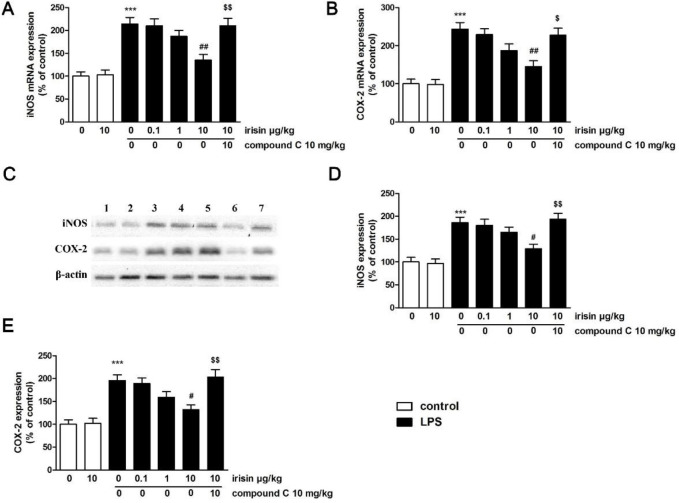
(A, B) Effects of irisin on iNOS and COX-2mRNA expression in the uterus. (C) Effect of irisin on iNOS and COX-2 protein expression in the uterus. Lane 1: sham group; Lane 2: sham + irisin (10 μg/kg, IV) group; Lane 3: LPS group; Lane 4-6: LPS + irisin (0.1, 1, 10 μg/kg, IV) treated groups; Lane 7: LPS + irisin (10 μg/kg, Iv) + compound C group. Protein levels of iNOS (C) and COX-2 (E) were normalized relative to the expression of β-actin. Values were the mean ± SEM (n=6 per group). *** *P*<0.001 vs the sham group; #*P*<0.05 and ## *P*<0.01 vs LPS group; $*P*<0.05 and $$*P*<0.01 vs LPS + irisin (10 μg/kg, IV) group

LPS administration led to increases in iNOS mRNA and protein expressions in the uterus (*P*<0.001 for mRNA and protein, Figures 5A, 5D). Irisin treatment at the concentration of 10 μg/kg inhibited LPS-induced abnormal expression of iNOS mRNA and protein in the uterus (*P*<0.01 and *P*<0.05). Similar increases were also observed in COX-2 expression after LPS treatment (*P*<0.001 for mRNA and protein, Figures 5B, 5E), and these enhancements were suppressed by irisin treatment (*P*<0.01 for mRNA and *P*<0.05 for protein). Interestingly, compound C co-treatment reversed irisin’s improvement effects on iNOS and COX-2 expression.

**Figure 6:**
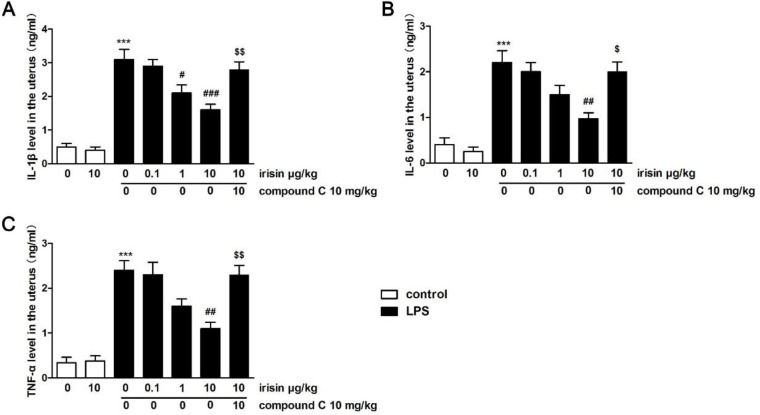
Effects of irisin on IL-1β (A), IL-6 (B), and TNF-α (C) expression in the uterus. Values were the mean ± SEM (n=6 per group). *** *P*<0.001 vs the sham group; ##*P*<0.01 and ### *P*<0.001 vs LPS group; $*P*<0.05 and $$*P*<0.01 vs LPS + irisin (10 μg/kg, IV) group

Similarly, inflammatory cytokines IL-6, IL-1β, and TNF-α were dramatically increased in the uterus stimulated by LPS (*P*<0.001 for IL-1β, TNF-α, and IL-6, Figure 6). The increases were reversed by 10 μg/kg irisin pretreatment. Compound C co-treatment abolished irisin’s improvement effects on these cytokines in the uterus (*P*<0.05 for IL-6, *P*<0.01 for IL-1β and TNF-α). 

***Irisin restores abnormal expression of oxidative stress markers in the uterus of LPS-treated mice***

**Figure 7:**
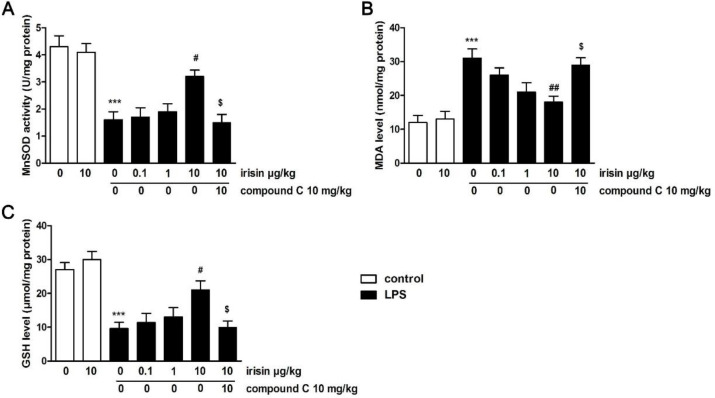
Effects of irisin on MnSOD activity (A), MDA (B), and GSH (C) levels in the uterus. Values were the mean ± SEM (n=6 per group). *** *P*<0.001 vs the sham group; #*P*<0.05 and ##* P*<0.01 vs LPS group; $*P*<0.05 vs LPS + irisin (10 μg/kg, IV) group

To further evaluate the influence of irisin on oxidative stress markers in the uterus of LPS-treated mice, we measured the concentrations of GSH, MDA, and MnSOD in the uterine tissue. As can be seen, the levels of MnSOD and GSH were reduced and MDA expression was enhanced after LPS stimulation (*P*<0.001 for GSH, MDA, and MnSOD, Figures 7A-C), while irisin abolished the above results (*P*<0.05 for MnSOD and GSH, *P*<0.01 for MDA). However, compound C eliminated the protective function of irisin against oxidative damage in the uterus (*P*<0.05 for GSH, MDA, and MnSOD).

## Discussion

In this research, results indicated that irisin possesses a protective effect on female mice with endometritis. The protective function of irisin was achieved by inhibition of inflammatory factors (i.e., IL-6, IL-1β, TNF-α, iNOS, and COX-2) through the AMPK-NF-κB pathway. In addition, irisin’s beneficial function was also related to its antioxidation, as evidenced by its regulation of abnormal MnSOD, GSH, and MDA levels.

Endometritis is characterized by congestion, edema, irregular shape, and poor toughness of the uterus (19). LPS injection is a commonly used method to trigger endometritis in animals (17). In our study, similar pathological features of endometritis appeared in the uterine tissue of mice injected with LPS. According to previous studies, inflammation and oxidative damage appear 12 hr and peaked 24 hr after LPS administration (20). Hence, tissue samples were collected 24 hr after LPS intrauterine perfusion in our research. Our study identified for the first time that LPS causes significant decreases of irisin in the serum and uterine tissue of mice. More importantly, 4 days of pretreatment with exogenous irisin revered LPS-induced decreases of irisin expression, as well as LPS-induced pathological changes in mice. 

Inflammation, which was regulated by NF-κB, the nuclear transcription factor, is involved in multiple diseases like atherosclerosis, cancer, myocardial infarction, kidney diseases (21), as well as endometritis (4, 16). Neutrophils are the major cells releasing various inflammatory cytokines, such as TNF-α, IL-1β, and IL-6 (22). These inflammatory factors are associated with the injury of the endometrium and disruption of epithelial integrity (17, 23). A previous study indicated that suppression of these inflammatory factors could improve LPS-induced endometritis (24). Furthermore, COX-2 and iNOS also play predominant roles in LPS-stimulated inflammation. Wu *et al*. found that down-regulation of COX-2 and iNOS can alleviate endometritis (25). In the present research, inflammatory mediators (i.e., IL-6, IL-1β, TNF-α, COX-2, and iNOS) were remarkably enhanced after LPS administration. However, the treatment of exogenous irisin reversed these alterations in the uterus, and the decreases of inflammatory mediators were accompanied by uterine tissue repairment as evidenced in the histological examination, suggesting irisin has a protective effect on endometritis by suppressing the inflammatory response. 

Numerous studies found irisin’s beneficial effect was achieved via regulation of the AMPK pathway (12, 26). In our study, LPS injection decreased AMPK expression in the uterus of female mice. Meanwhile, NF-κB, a key element in AMPK signaling, was increased in the uterus of LPS mice. However, irisin treatment up-regulated AMPK expression and inhibited NF-κB activation. Intriguingly, when compound C was used, irisin’s effect on AMPK and NF-κB expression was abolished. Meanwhile, the irisin-decreased levels of IL-1β, IL-6, TNF-α, COX-2, and iNOS were also reversed by compound C. Given that gene transcription of inflammatory mediators IL-1β, IL-6, TNF-α, COX-2, and iNOS was induced by NF-κB activation (11), our results suggested that the positive effect of irisin on LPS-induced inflammation in the uterus was realized by regulating the AMPK-NF-κB pathway. 

iNOS is a pleiotropic molecule, involved not only in inflammation but also in oxidative stress (27). The iNOS expression in the uterus was influenced by irisin treatment in our study. In order to explore whether irisin exerts antioxidant function on LPS-induced endometritis in the uterus, we detected the levels of direct oxidative stress markers GSH, MDA, and MnSOD in the uterine tissue. MDA, the ultimate product of lipid peroxidation, is a reliable marker for peroxidation (28). GSH and MnSOD are important endogenous antioxidants that can scavenge free radicals to exert antioxidant functions (28). Consistent with previous studies (29, 30), increased expression of MDA and decreased expression of GSH and SOD were induced by LPS in our study. Interestingly, irisin reversed these abnormal states. These data confirmed that irisin’s improvement of endometritis was associated with its antioxidant ability. Whereas the associated mechanism needs to be disclosed by more data.

 To the best of our knowledge, this is the first time irisin’s protective effect on LPS-induced endometritis in mice is reported. This protective effect of irisin was achieved by reducing inflammatory factors such as TNF-α, IL-1β, IL-6, COX-2, and iNOS by regulating the AMPK-NF-κB pathway. This does not mean that we deny that the protective function of irisin may also be related to other signaling pathways such as the autophagy pathway, apoptosis pathway, and other AMPK-related pathways. We hope that the positive effect of irisin on uterus can be fully understood in the future, after all, research focusing on irisin’s function on uterine diseases is rare.

## Conclusion

Our study indicated that irisin can ameliorate LPS-induced inflammation in the uterus of female mice, and improved function of irisin was related to regulation of the AMPK-NF-κB pathway. Based on our data, irisin is a potential candidate for clinical endometritis treatment. Nevertheless, more data are needed to confirm its function. 

## Authors’ Contributions

XY and XJ designed the study. YH, YZ, JC, CS, ZC, CJ, LC, LX, FL, and WN conducted the experiment and analyzed the data. XJ wrote the manuscript draft and XY made the revision. All authors read and approved the final manuscript.

## Conflicts of Interest

All authors declare that they have no conflicts of interest.
